# LEAN: Real-Time Analysis of Resistance Training Using Wearable Computing

**DOI:** 10.3390/s23104602

**Published:** 2023-05-09

**Authors:** William Coates, Johan Wahlström

**Affiliations:** 1 Independent Researcher, Bath BA1 2TP, UK; 2 Department of Computer Science, University of Exeter, Exeter EX4 4QF, UK

**Keywords:** wearable computing, mobile sensing, exercise form classification, repetition counting

## Abstract

The use of fitness apps to track physical exercise has been proven to promote weight loss and increase physical activity. The most popular forms of exercise are cardiovascular training and resistance training. The overwhelming majority of cardio tracking apps automatically track and analyse outdoor activity with relative ease. In contrast, nearly all commercially available resistance tracking apps only record trivial data, such as the exercise weight and repetition number via manual user input, a level of functionality not far from that of a pen and paper. This paper presents LEAN, a resistance training app and exercise analysis (EA) system for both the iPhone and Apple Watch. The app provides form analysis using machine learning, automatic repetition counting in real time, and other important but seldom studied exercise metrics, such as range of motion on a per-repetition level and average repetition time. All features are implemented using lightweight inference methods that enable real-time feedback on resource-constrained devices. The performance evaluation includes a user survey and benchmarking of all data science features using both ground-truth data from complementary modalities and comparisons with commercial apps.

## 1. Introduction

With at least 2.8 million people dying each year as a result of being overweight or obese [1], fitness apps are becoming increasingly important to encourage regular physical exercise [2] and monitor physiotherapy [3]. One study found that the use of smartphone fitness apps was associated with both increases in physical activity and significant reductions in body weight and body mass index [4]. Many outdoor cardio activities can be tracked with relative ease using a device with an embedded global navigation satellite system (GNSS) receiver [5,6]. However, creating an app that automatically tracks resistance training is much more difficult. One factor that makes this so challenging is the large number of commonly practised resistance training exercises. Moreover, most of these exercises are typically performed indoors with little variation in position, meaning that GNSS information is largely useless. As a consequence, the majority of current resistance exercise tracking apps are not nearly as enticing in terms of ease of use, automatability, and simplicity as the corresponding cardio tracking apps. Most require tedious interaction during a workout, such as manually entering the number of repetitions completed.

One overlooked feature that would be of great benefit in resistance exercise tracking apps is form analysis. Automatic form analysis would, for example, greatly reduce the risk of injuries. Research on CrossFit training has found that anywhere between 19.4% [7] and 73.5% [8] of people who regularly participate in CrossFit training have sustained an injury during training sessions. Moreover, Gray et al., in 2015, indicated that 36.2% of injuries in the gym are caused by “overexertion/strenuous/unnatural movement” [9]. In addition to reducing the risk of injuries, good form will also result in greater muscle gain over time [10]. Moreover, automatic form analysis could also help beginners overcome so-called gymtimidation, that is, anxiety about making mistakes in the gym [11]. One way to learn and practice proper exercise form is to hire a personal trainer. However, on top of a gym membership, the cost of a personal trainer is too expensive for many. Another alternative is to watch online video tutorials. The drawback here, however, is that there is no feedback mechanism. It is not always possible to evaluate your own form in certain exercises, and users may think that they are performing an exercise correctly even though they are not. LifeSmart claims to be the first wearable to provide exercise form feedback using unsupervised learning methods [12]. The form analysis system assumes that the first few repetitions of a set are completed with good form. Subsequent repetitions in the same set are evaluated against that reference to assess whether the user is maintaining good form throughout the set. Since our exercise system is designed to also be useful for beginners, the assumption that the first few repetitions are performed with good form cannot be made, and therefore, an unsupervised learning approach would not be suitable. A more common approach for form analysis is with the use of supervised learning models. W8-Scope proposes a resistance training monitoring system using a 3-axis accelerometer and 3-axis magnetometer mounted on top of the weight stack of a cable machine [13]. The main drawbacks of this approach are the increased cost and inconvenience related to using pre-installed sensors. More generally, there is a need for form analysis systems that do not require multiple sensors mounted on the body [14,15]. The form analysis presented in this paper can be implemented using only measurements from off-the-shelf smartwatches, thereby both obviating the need for pre-installed sensor infrastructure and making it possible to analyse any exercise involving sufficient wrist movement.

**Figure 1:**
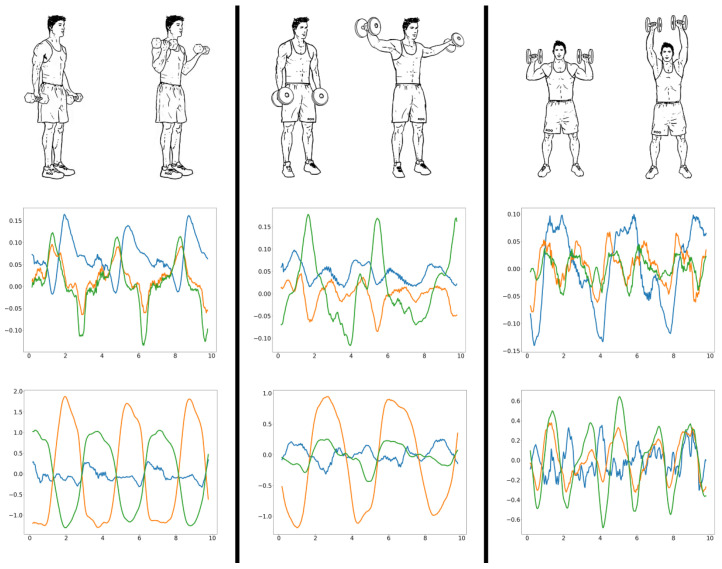
Illustration of the three resistance training exercises considered in this paper with motion graphs of 10 s of acceleration (m/s${}^{2}$) and rotation rate (rad/s) in the middle and bottom rows, respectively. The x, y, and z dimensions are displayed as blue, orange and green, respectively.

Repetition counting takes advantage of the repeating motion patterns seen during an exercise set with multiple repetitions (see Figure 1). A change in the repetition phase (eccentric and concentric) of the exercise is represented by peaks and troughs in the inertial signal of interest [16,17]. Most studies on repetition counting start with a pre-processing step to smooth and remove the noise typically found in inertial data. This is commonly achieved using some form of a low-pass filter [18]. Next, dimension reduction is applied to the three-axis inertial data. A naive solution is to simply hard code the major dimension for each exercise [19]. However, a better approach is to dynamically select the dimension with the highest variance [20]. This dimension is most likely the predominant exercise direction and will produce the most accurate results when counting peaks or troughs. Since the repetition count can be calculated using either the peaks or troughs of inertial data, many proposed solutions simply select either peaks or troughs for counting all exercises. MiLift [21], which achieves an average error of 1.12 reps (out of an average of 9.65), utilizes dynamic decision-making based on the spikiness of the peaks and troughs by calculating the vertical displacement over a small time window. A novel, alternative approach to repetition counting uses a convolutional neural network to identify if a window of motion data contains the start of a repetition [22]. As this is a binary classification, motion window sizes are varied across different exercises to ensure that a motion window cannot contain more than one start of a repetition. One drawback of this approach, however, is that individual neural networks must be trained separately for each type of exercise. Moreover, many methods for repetition counting compute the number of repetitions after the exercise set, or entire workout, has been completed [19,21,22,23]. In this study, we instead present a repetition-counting algorithm specifically designed to enable real-time feedback on devices with limited memory resources, such as smartwatches.

**Figure 2:**
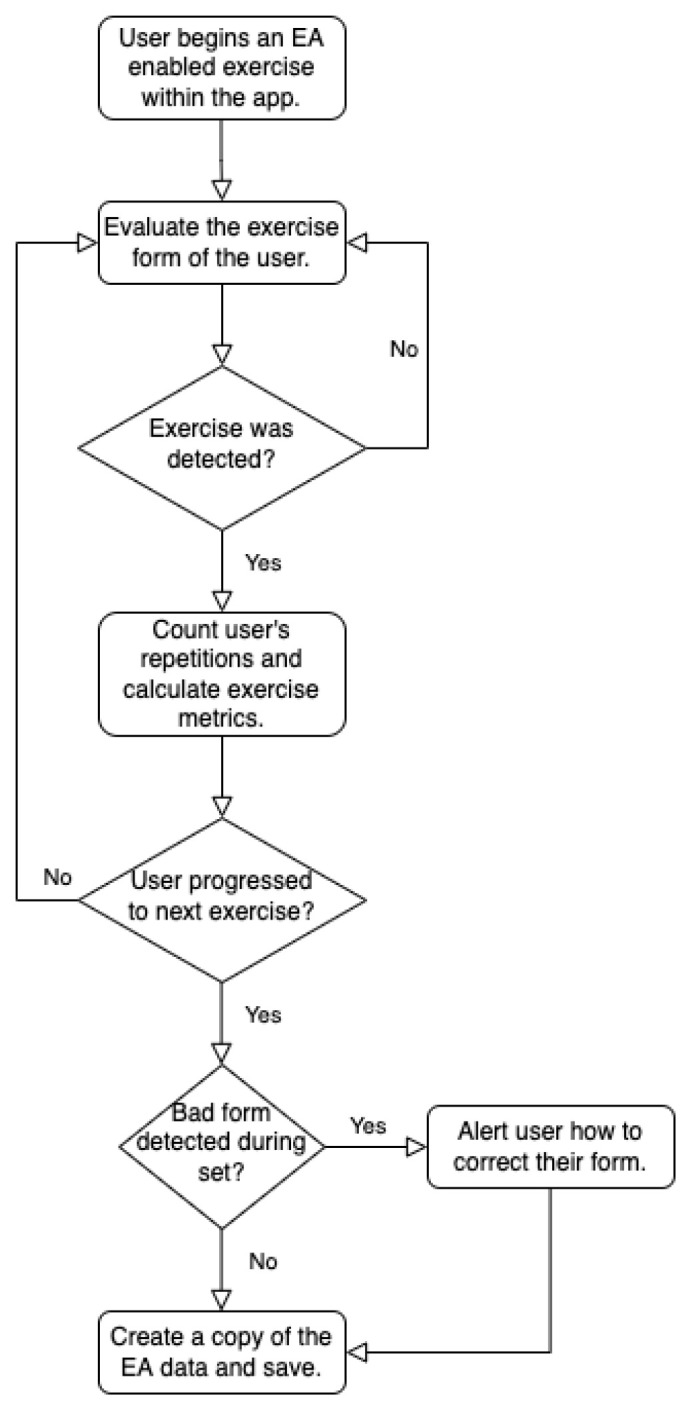
Process diagram illustrating the relationship between the implemented EA features.

In summary, despite the large number of available resistance training apps, they typically (i) only provide a very limited number of EA features; (ii) do not allow for real-time feedback; and (iii) sometimes utilize computationally complex and privacy-invasive sensors, such as cameras. This paper presents the resistance training app LEAN (Lightweight Exercise tracker for Athletes and Novices) that contains a large range of EA features and real-time feedback, all based on convenient measurements from a smartwatch. The app is available for the iPhone with a companion app for the Apple Watch. In addition to form analysis, LEAN also provides automatic repetition counting and exercise metrics such as the range of motion on a per-repetition level. The relationship between these EA features is illustrated in Figure 2. While it is possible to design EA systems using multiple IMU sensors, this presents a barrier to entry both economically and in terms of usability [24]. Given that this paper focuses on designing an EA for a broad set of users, convenience and ease of use are important factors. For this reason, this paper will collect measurements from a single IMU embedded in an Apple Watch remaining on the wrist of the user.

### Contributions

The main contributions of this paper are:

- Form classification that does not assume that the first few repetitions during an exercise set are of good form, does not place the inertial sensors on the weight stack, and does not require multiple sensors mounted on the body.
- Integration of the form analysis classification model and the repetition-counting algorithm to improve the computational efficiency of the system.
- An incremental repetition-counting algorithm that uses dynamically sized buffers to count repetitions in real time and that does not have to store long time periods of motion data in memory.
- A description of how to compute fine-grained exercise metrics. This includes the ratio of the time spent on concentric (shortening the muscle) and eccentric (lengthening the muscle) motions and the range of motion.

All in all, we present an EA system that is efficient enough to be used in a smartwatch for extended periods of time while automatically tracking resistance training metrics at a level of detail similar to existing cardio tracking apps.

Section 2 describes the EA algorithms, Section 3 describes the front-end app design, Section 4 presents the performance evaluation, and Section 5 concludes the paper.

## 2. Exercise Analysis

In this section, we describe the inference algorithms for exercise classification, form classification, repetition counting, estimating timing statistics, and estimating range of motion.

### 2.1. Exercise and Form Classification

The exercise and form classifiers need to be able to run over an extended period of time with modest energy requirements, while at the same time making minimal assumptions on the experience or characteristics of the user. To satisfy these requirements, we decided to utilize supervised, lightweight machine learning algorithms. In particular, we used a gradient-boosted classifier, which, similarly to a random forest classifier, combines multiple decision trees to build a more accurate model.

To analyse a user’s form, independent machine learning models were created for each exercise. The chosen exercises were bicep curls, lateral raises, and shoulder presses. All models included a “Good” and “Other” label. “Good” indicates the user is exercising with good form, and “Other” indicates the user is not currently exercising. Thereby, each model can be used to indicate both when the user is currently exercising and the state of their current form. One challenge with the “Other” label is that the motion of a user in between exercise sets is random and diverse. The user could be sitting still, walking around, or racking their weights. To overcome this problem, the training data for the classification of “Other” is a mixture of all these actions. Additional labels classify the user as exercising with a certain type of poor form, such as performing the exercise with bad range or performing the exercise too fast. The data captured during training includes the rotation rate, acceleration, quaternion, and gravity (all part of watchOS).

**Figure 3:**
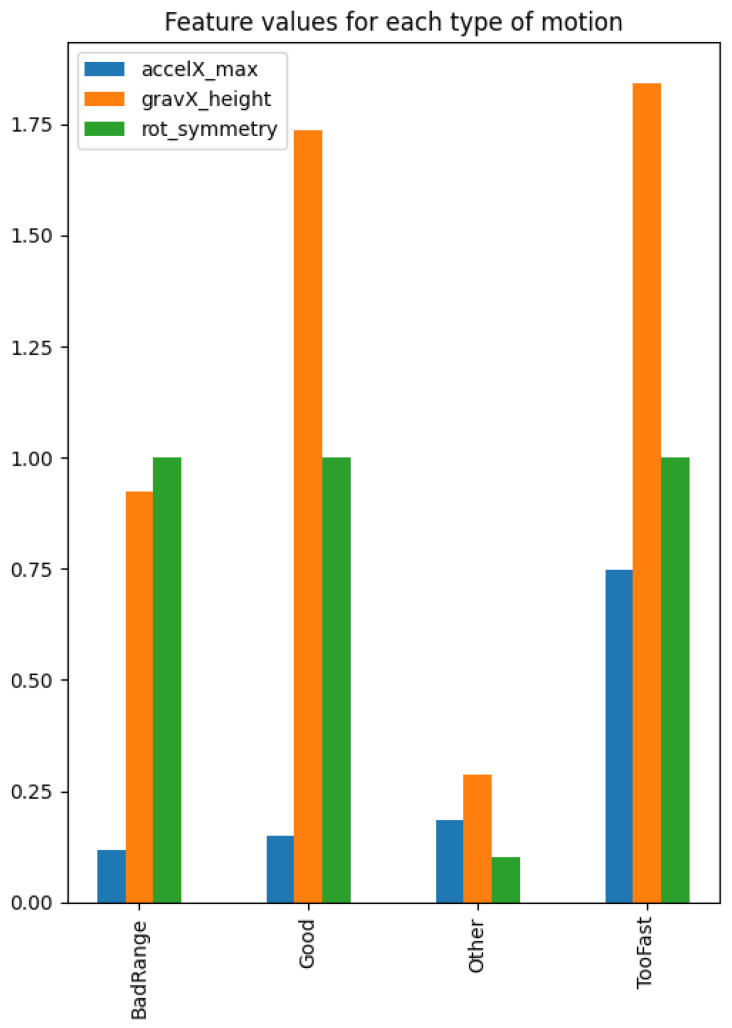
The average training and testing values of features extracted for the bicep curl.

All recorded data were annotated with the exercise performed in that particular recording. The classification was based on 4 s time windows with 50% overlap. This window length is long enough to capture the motion of at least one repetition but short enough to enable the detection of a change in form or exercise relatively quickly. A 50% overlap further improves the responsiveness of the model by enabling form analysis every two seconds rather than every four. The next step was to extract features from these windows. For the bicep curls and the lateral raises, the exercise and variations of form could be identified by visually inspecting motion data, enabling us to extract a minimal number of manually selected features, as outlined in Table 1. Each feature was responsible for signalling a specific classification or motion characteristic of the exercise. For instance, during a bicep curl, the rotation rates in the Y and Z dimensions almost always intersect at Y=0, as shown in Figure 1. This occurrence was represented using a binary feature by summing the number of rotation rate Y, Z pairs, where one value is positive and the other is negative. If this sum exceeds 87.5% of the total number of rotation rate Y, Z pairs in a window, the feature is set to “1”, signalling that a bicep curl is occurring. Figure 3 shows the average feature values for each bicep curl form label.

Additional manually extracted features included the following: the maximum acceleration of a specific axis to indicate an exercise is being performed too fast; the difference between the maximum and minimum gravity value of a specific axis to indicate an exercise is being performed with bad range; and the total number of turning points of a gravity or attitude axis to indicate an exercise is being performed at all [25]. Usually, a lower number of turning points are found when the user is currently exercising. By fixating the axes from which we extract features, we assume that the Apple Watch always has the same orientation with respect to the wrist. To relax this assumption, the app allows the Apple Watch to be worn in two different orientations and on either of the user’s wrists. This is made possible by two settings within watchOS’s user settings: one setting to indicate which wrist the Apple Watch is worn on and one setting to set the orientation of the watch. These settings automatically adjust the display orientation and adjust the inertial axes to accommodate all common orientations.

Classification using a small number of manually extracted features worked very well for exercises that include a rotation of the wrist. However, for the shoulder press, the motion of the wrist is primarily a translation in the vertical direction. In other words, the rotation rate is mostly noise and gives no indication of the current activity of the user, which makes the form classification much more challenging. Therefore, the feature extraction approach taken for the shoulder press was to extract as many features as possible from the acceleration data in both the time domain and frequency domain. Taking inspiration from a time series feature extraction library [26], we calculated the following statistical features for each axis: acceleration, mean, standard deviation, average absolute difference, minimum, maximum, minimum–maximum difference, median, median absolute deviation, interquartile range, number of values above the mean, number of local maxima, skewness, kurtosis, energy, average resultant acceleration, and signal magnitude area. Additionally, we converted the acceleration data from the time domain to the frequency domain via a fast Fourier transform and calculated the same statistical features, totalling 94 features altogether. To reduce the number of features to be computed, we attempted to use univariate feature selection. This revealed that the feature “skewness” had the least influence on the model’s classification. However, removing this feature resulted in a clear reduction of the model’s F1-score, so all 94 original features were kept. In the ‘Just Workout” mode, the app first uses exercise classification to identify the exercise being performed and then start counting repetitions and analyze the form of that particular exercise. The exercise classification in this mode used the same features as the form classification.

Given the large number of features, a test was conducted to identify how the efficiency of the Apple Watch app compares to the official Apple Workout app. To create a worst-case scenario in terms of efficiency, a modified version of LEAN was created. In this modified version, the shoulder press form analysis model (94 features extracted every 2 s) runs for the entire workout session. With this implementation, three workout sessions of 70 min caused the battery to drop an average of 10%. We then ran three “Traditional Strength Training” 70-minute workouts in the Apple Workout app on the same Apple Watch. This caused an average drop in battery of 11% on the same Apple Watch. All workouts started from a battery life of 60% in a controlled temperature environment. Therefore, we can conclude that LEAN is at least equally efficient as the official Apple workout app for the Apple Watch, despite being tested in a worst-case scenario.

### 2.2. Repetition Counting

**Figure 4:**
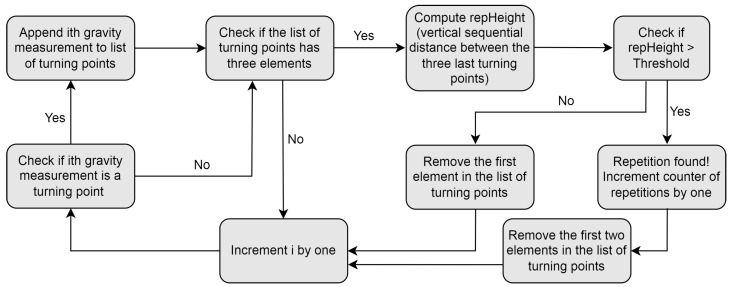
Program flow chart for the repetition-counting algorithm.

**Figure 5:**
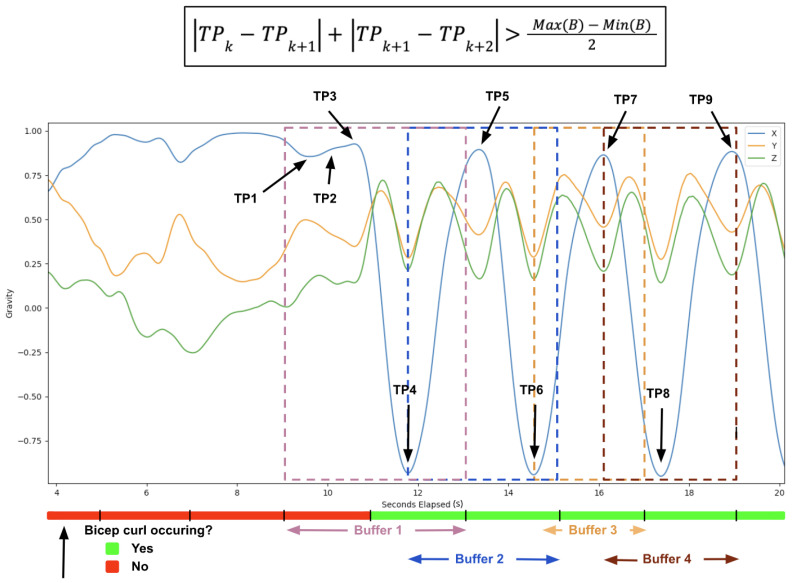
A gravity plot of before and during a set of bicep curls with repetition-counting algorithm buffers and turning points indicated.

The repetition-counting algorithm counts the user’s repetitions during an exercise set and stores the start, middle (when the motion has only been performed in one direction) and end timestamps for each repetition. These timestamps are used to calculate exercise metrics, such as the average repetition time and the ratio of time spent in the concentric and eccentric phases of each repetition. The algorithm only runs when the exercise form classification model detects that the user is currently exercising. This helps to reduce the risk of false-positive repetitions that many other papers have to locate and remove via various heuristic methods [23]. The algorithm is detailed in Algorithm 1 and Figure 4 and is also illustrated in Figure 5. The two inputs of the algorithm are the gravity buffer and an array of the current indices of turning points, for which it has not yet been determined whether they are part of a repetition or not. In the algorithm, a turning point is defined as being a data point where the two consecutive values before and after the data point are both either larger or smaller than the value of the data point itself. In accordance with [21], the signal used to count repetitions is the gravity acceleration vector provided by the Apple Watch. Further, the algorithm is applied to the axis with the largest variance, as this is likely the dominant motion direction during the exercise and will produce the most accurate repetition count.
**Algorithm 1** Repetition counting algorithm**Input:** B = Gravity buffer array; TP = Turning points array **Output:** C = Repetition count      *Initialisation*:   1:$\begin{array}{l} C\leftarrow 0\\ LOOP\;Process \end{array}$  2:**for** i←0 to B.length **do**  3:   **if** (isTurningPoint(B[*i*])) **then**  4:     TP.append(B[*i*])  5:   **end if**  6:   **if** (TP.length==3) **then**  7:     height1←abs(TP[0]−TP[1])  8:     height2←abs(TP[1]−TP[2])  9:     repHeight←height1+height210:     threshold←(Max(B)−(Min(B))/211:     **if** (repHeight>threshold) **then**12:        C←C+113:        TP←TP.removeFirst(2)14:     **else**15:        TP←TP.removeFirst(1)16:     **end if**17:   **end if**18:**end for**19:**if** (TP.length>0) **then**20:   B←B.removeFirst(TP[0])21:**else**22:   B←B.removeAll()23:**end if**24:**return** *C*

### 2.3. Other Exercise Metrics

The calculation of exercise metrics relies upon data output by the repetition-counting algorithm. For example, the total exercise set time can be calculated as the difference in time between the start of the first repetition and the end of the last repetition. Moreover, it is also straightforward to compute other exercise metrics, such as the average repetition time and average time spent in concentric and eccentric motion. This extends prior studies that have primarily focused on metrics such as the length and timing of repetitions and energy expended by the arm during an exercise [27,28,29]. The range of motion is defined as the angle through which the Apple Watch is rotated on the most dominant axis during either the concentric or eccentric phase of a repetition. Research has shown that using a full range of motion rather than a partial range of motion helps to maximise muscle strength [30]. This metric is only calculated for the bicep curl and lateral raise, both of which exhibit meaningful rotation, unlike the shoulder press. To compute this metric, we first store the roll angles measured during a repetition in an array. The sum of the differences between each consecutive local turning point within this array is equal to the total angle rotated during the repetition. This value is then halved since a repetition consists of the same motion repeated twice, in opposite directions. Due to the consistent orientation of the Apple Watch on the wrist, we can safely assume that roll will be the correct attitude axis to measure the range of motion with. However, other exercises may require combining multiple Euler angles due to the varied orientation of the wrist itself during the exercise.

## 3. App Design

In this section, we describe the design of the apps that incorporate the exercise features presented in Section 2. The final result is a resistance training iPhone app with an optional Apple Watch app. Workouts (i.e., predefined sequences of exercises) created on the iPhone automatically sync to the user’s Apple Watch. The apps contain 20 popular resistance exercises by default, with the possibility for users to add new exercises. The apps support both traditional repetition exercises, where the exercise is completed by repeating a specific motion, and isometric exercises, where a specific body position is held for a period of time. Three of the included exercises (bicep curl, lateral raise, and shoulder press) have EA features enabled. During an exercise set with an EA-enabled exercise, the Apple Watch will vibrate when the user completes the penultimate repetition, signalling that they have one repetition remaining. For these exercises, the user can also view detailed statistics on their performance over each set. These include the average repetition time, the average range of motion, the percentage of time exercising, and the average time spent in eccentric and concentric motion expressed as a percentage of the full repetition.

### 3.1. iPhone App Front-End

**Figure 6:**
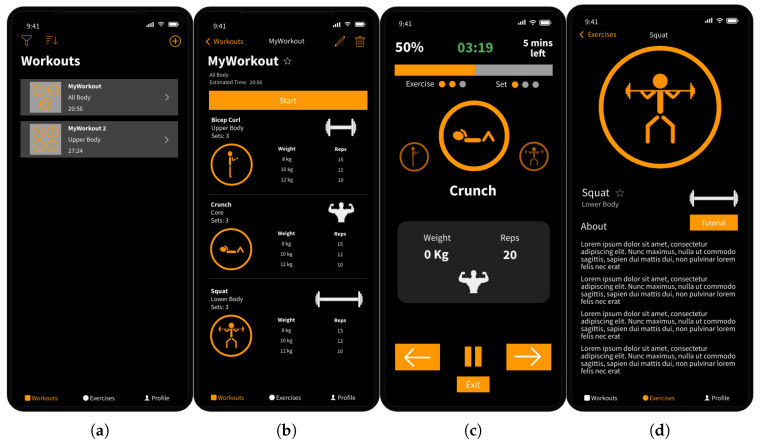
Wireframes of the iPhone interface: (**a**) workout list page, (**b**) workout detail page, (**c**) working out page, and (**d**) exercise detail page.

Generally, the iPhone user interface is kept as simple as possible. The iPhone app is divided into three main views, navigated by a tab bar at the bottom of the display. The first tab, named “Workouts”, displays a list of all workouts created by the user, as shown in Figure 6a. From this page, the user can perform three main actions: they can sort, filter, and search for workouts in the workout list; press on a workout in the list to view more details; or press on the plus button to create a new workout. The user interface uses images to represent different exercises, and each workout is presented as a preview of the exercises contained within it. Similarly, images are also used to indicate what equipment is required for each exercise. When pressing on a workout from the list, a page containing more details about the workout is displayed, as shown in Figure 6b. From here, the user can either start, edit, or delete the workout. Below the “Start” button is the list of exercises included in the workout, including the weight and repetition target for each set. Each exercise within the workout has a “sets” option. This allows the user to create a workout with consecutive exercise sets with different weight and repetition targets more easily.

Figure 6c shows the page displayed during a workout. The top of the page indicates progress through the workout, expressed as a percentage, estimated time remaining, a progress bar, and indicators of the current exercise and set. The most important information is displayed in the centre of the display, detailing the current exercise along with the weight, target repetition count and the equipment required. Additionally, images of the previous and next exercises are displayed with reduced opacity on either side of the current exercise image. The workout controls are situated at the bottom of the display for easier reachability on a mobile device. The “Exit” button allows the user to exit the workout early before completing all exercises, and the “play/pause” button allows them to stop and start the workout timer. Lastly, the arrow buttons allow them to move to the next or previous exercise set. Moving to the previous exercise was included for two main reasons. First, the user may accidentally double-tap the next exercise button and need to go back. Secondly, it may not always be possible to complete a workout in the intended order when a gym is busy with a large proportion of equipment or machines already occupied. This functionally allows the user to skip an exercise and come back to it later on when the equipment or machine becomes available.

The second tab, named “Exercises”, has a similar layout and functionality as the “Workouts” tab. In particular, the tab allows the user to sort, filter, and search the list of exercises; press on an exercise in the list to view more details; or press the plus button to add a new exercise. The “Exercise Details” page, shown in Figure 6d, displays information such as which muscles the exercise utilises, along with a button that takes you to a YouTube tutorial of the correct form for that particular exercise. The last tab, named “Profile”, contains a list of completed workouts, user statistics, and a settings page. The list of completed workouts includes workouts completed on both the iPhone and the Apple Watch, enabling the user to gain more detailed insight into the EA features measured by the Apple Watch on a larger display.

### 3.2. Apple Watch App Front-End

For a smartwatch, it is even more important to keep the interface as simple as possible, as it has a much smaller display. The main view will display a simplified version of the “Workouts” tab on the iPhone. Since the watch’s display is so small, the user will not be able to create or edit workouts from the Apple Watch. While it would technically be possible to include this functionality, the interface would have to be overly complex.

**Figure 7:**
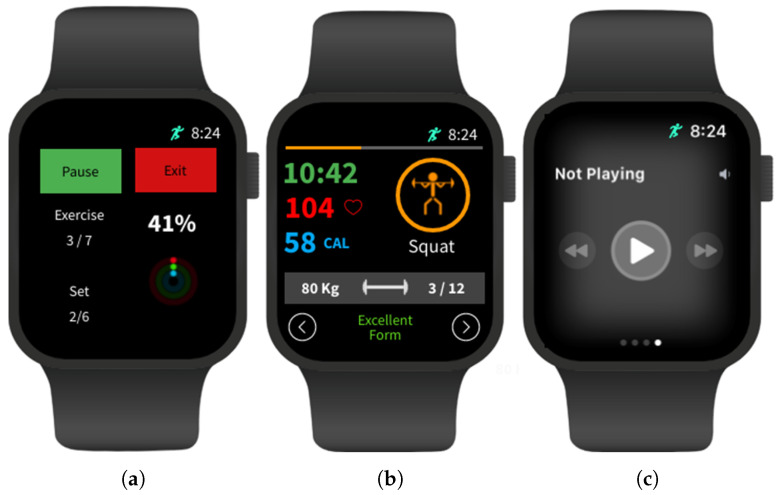
Wireframes of the Apple Watch “Working Out” interface: (**a**) secondary workout page, (**b**) default page, and (**c**) audio control.

The “Working Out” view was split into the three pages shown in Figure 7. The user can swipe between them during a workout, with Figure 7b being the default. This page displays the information and controls most important to the user during a workout. For exercises that have EA features enabled, the text at the bottom of the display will show the current prediction of form or “Resting” if the user is currently not performing an exercise. In addition, the repetitions target displayed in the grey rectangle will be expressed as a fraction of the current number of repetitions completed. Figure 7a will act as a secondary workout page, displaying secondary information and controls. This includes pausing and exiting the workout early, as well as the current exercise and set number the user is performing. Figure 7c is made available by Apple to view and control the audio currently playing, allowing the user to easily control their music without having to leave the app.

## 4. Performance Evaluation

This section demonstrates the performance and utility of the LEAN app and its included EA features. The code and the data for building the app and for recreating the performance tests presented in this section are available at https://github.com/Resistance-Training-App (accessed on 1 March 2023).

### 4.1. Data Collection

**Figure 8:**
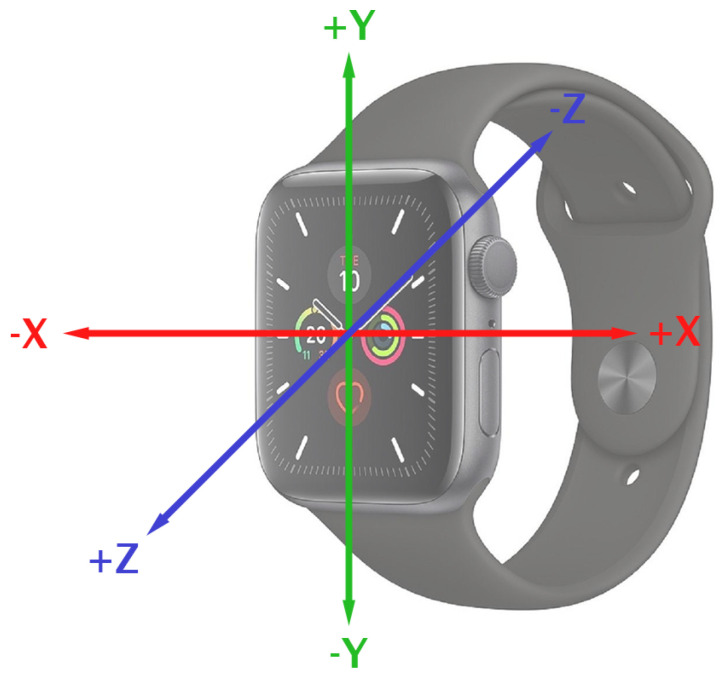
Sensor axes of the Apple Watch Series 5 used for data collection.

When recording training data, we ensured that the exercise and form classes were balanced by recording exactly 20 min of data per exercise form and classification label. A single user recorded the training data for all exercises and all exercise forms (a larger number of users was considered in the user survey in Section 4.5) with a lightweight dumbbell during two-minute recording sessions. This ensured that the motion of the wrist would be similar to when using a heavier weight whilst being able to comfortably perform two minutes of the exercise with no change in exercise form. All training data were recorded at a frequency of 40 Hz. Based on previous studies, this sampling rate allows for a good trade-off between accuracy and resource usage [31]. During data recording, the Apple Watch was worn with the digital crown facing towards the hand, on the left wrist. The three spatial axes in relation to the Apple Watch are shown in Figure 8.

### 4.2. Exercise and Form Classification

**Figure 9:**
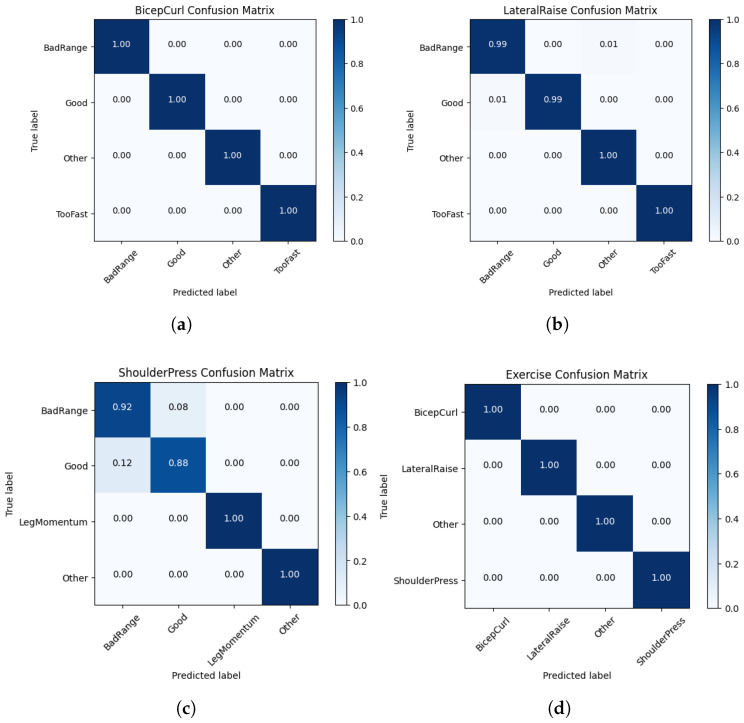
Confusion matrices of form analysis models for (**a**) the bicep curl, (**b**) lateral raise, and (**c**) shoulder press, and (**d**) confusion matrix for exercise classification.

The form classification results are presented in Table 2 and Figure 9a–c. Using an 80:20 train–test split, the form analysis models for the bicep curl and lateral raise produce a recall of 99% to 100%. Furthermore, the app allows the testing of these models using real-time Apple Watch motion with the prediction and confidence displayed on the iPhone. Use of this app confirms the high accuracy of these models, with a few anomalies occurring mostly during the transitions between two activities. The shoulder press form analysis model had some difficulty in separating between a good shoulder press and a shoulder press with bad range. As shown in Table 2, the recall was 88% and 92% for a good shoulder press and a shoulder press with bad range, respectively. This decrease in performance can be explained by the similarity of the wrist acceleration during these two motions. In most situations, the shoulder press form classification works well. However, sometimes when transitioning from a set of good shoulder presses to being stationary, a single classification of “bad range” could occur in between the classifications of “good” and “other”.

In the “Just Workout” mode, we first identify the exercise the user is performing before switching to the form analysis model of that particular exercise. As shown in Figure 9d, the recall of all classification labels for this model was 100%. This can be explained by the fact that the considered exercises are composed of very distinct motions. The accuracy can be expected to degrade when including multiple exercises with similar wrist motions, such as the shoulder press and bench press.

### 4.3. Repetition Counting

**Figure 10:**
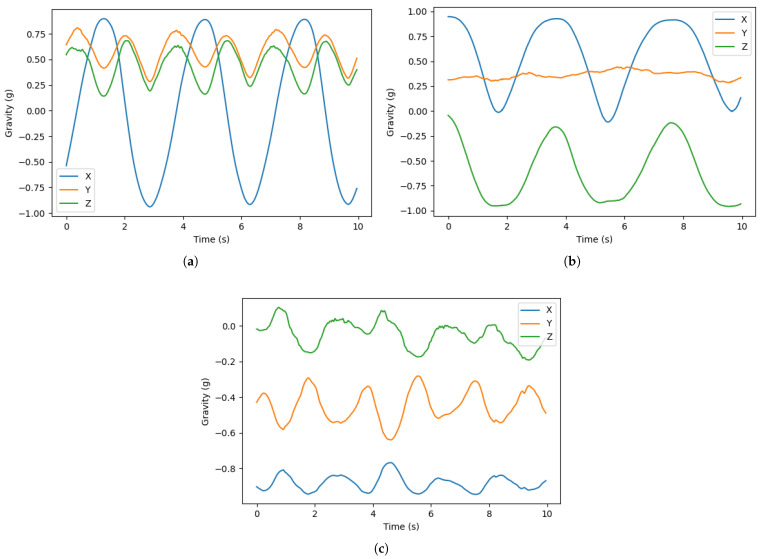
A total of 10 s of gravity data demonstrating good form of (**a**) bicep curls, (**b**) lateral raises, and (**c**) shoulder presses.

An existing app that is available for both the iPhone and Apple Watch is Gymatic Workout Tracker (GWT), developed by Vimo Labs. It claims to be the first and only app that automatically identifies exercises and counts the user’s repetitions. The repetition-counting algorithm in LEAN, described in Section 2.2, was compared with the repetition counting feature in GWT. The test was conducted using two Apple Watches side by side on the same wrist to ensure that both apps receive similar inertial data input. Three exercises were performed, each with three sets of 8, 10, and 12 repetitions. The results are displayed in Table 3. In total, LEAN miscounted twice and GWT miscounted three times, with all incorrect repetition counts missing one repetition. The incorrect repetition counts for our app only occurred with the shoulder press, the only exercise tested that does not involve a meaningful rotation of the wrist. As shown in Figure 10, a bicep curl or a lateral raise repetition can be much more easily identified using gravity compared to a shoulder press, where oscillations are not as consistent and amplitudes are smaller.

### 4.4. Other Exercise Metrics

**Figure 11:**
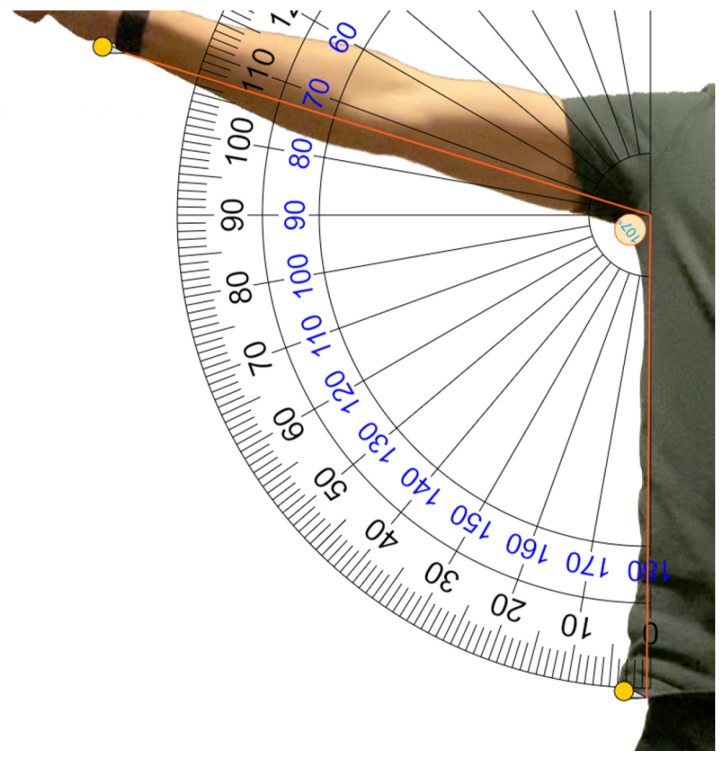
Estimating the range of motion in degrees of a lateral raise using frames from video footage.

To evaluate the accuracy of the exercise metrics, we video recorded one participant performing 10 repetitions of each exercise while running a workout on the Apple Watch at the same time. Using the video footage, it was possible to manually calculate precise estimates of the average repetition time and the eccentric/concentric ratio. The range of motion was more difficult to accurately estimate using video footage; one example of how this metric was estimated is demonstrated in Figure 11. In Table 4, these estimated metrics are compared to the metrics calculated by LEAN. The average repetition times of all three exercises calculated by the app were all within a tenth of a second of the times estimated using video footage. Moreover, the ranges of motion of bicep curl and lateral raise were within 4 degrees of estimations using video footage. With this level of accuracy, the exercise metrics provide highly valuable and detailed information to users, information which would have been extremely cumbersome or expensive to collect by other means.

### 4.5. User Survey

A beta version of the LEAN app was distributed to six volunteers via Apple’s beta distribution system, “TestFlight”. Out of the six volunteers, four owned an Apple Watch, with three being able to test the EA features, while the remaining volunteers only tested the iPhone app. The volunteers were instructed to perform a pre-made workout three times. The workout consisted of the three EA-enabled exercises: bicep curls, lateral raises, and shoulder presses, each with ten repetitions. For the first workout, the volunteers were asked to choose light weights that they could easily complete the workout with, while for the second workout, they were asked to select weights that would challenge them to complete each exercise set. The third workout was performed with what each volunteer would consider as bad form with light weights to reduce the risk of injury.

Table 5 presents the complete results. During the first workout, only one out of nine form analysis results from the three volunteers was reported as bad form, a lateral raise detected as having bad range. It is unclear whether this was a misclassification or the volunteer was indeed performing the exercise with insufficient range. The only repetition count that was incorrect was an underestimation by one repetition during a shoulder press. For the second workout, two form analysis results reported bad form, both during lateral raises. Since volunteers used heavier weights for this workout, it is suspected that these bad range classifications were accurate, as this is typical for lateral raises with heavier weights. Three repetition counts were underestimated by one during workout two, one for lateral raises, and two for shoulder presses. The increased miscounts may be due to the use of heavier weights, which could cause pauses or slow movements during the exercise. During workout three, all but one volunteer’s description of how they performed bad form matched with the result of the form analysis.

We also conducted a user survey to gather feedback on the volunteers’ experience with the app. The main results of the survey are summarized in Table 6. Overall, the feedback was positive. All respondents agreed or strongly agreed that each app was easy to understand and navigate. Three out of four respondents either agreed or strongly agreed that the form analysis was useful, while the remaining respondent neither agreed nor disagreed. Feedback for the repetition counting was slightly more mixed, with one respondent disagreeing that it was useful. When asked to elaborate, they stated, “I can count reps in my head fine.” This response may make perfect sense for those who are not interested in keeping track of their workouts in the long term. However, the primary benefit of automatic repetition counting is to alleviate the user’s burden of manually logging repetition counts after each exercise set. In addition to the questions shown in Table 6, we asked participants whether they would use the app in the future, with possible answers being “Yes”, “Maybe”, or “No”. Four out of six respondents answered “Yes”, while the remaining respondents answered “Maybe”.

## 5. Conclusions

This paper presented LEAN, an easy-to-use workout app for the iPhone and Apple Watch that provides analysis of resistance training at a level similar to cardio-based workout tracking apps. The apps were designed with simplicity in mind and deliver a familiar and native iOS and watchOS experience. In addition, they provide multiple exercise features, including form classification, repetition counting, and computation of exercise metrics, such as the average repetition time and the range of motion. One major contribution is the integration of form classification and repetition counting. This reduces both the number of false positives in the repetition counting and the computational complexity. In addition, a repetition-counting algorithm was implemented using an incremental buffer-based approach. This reduces memory requirements and enables real-time feedback, for example, smartwatch vibrations during the penultimate repetition to signal that only one repetition is remaining. The integration of multiple inference algorithms is taken one step further with the “Just Workout” mode. In this mode, the app first identifies the type of exercise being performed before starting to count repetitions and switching to the form analysis model of that particular exercise. During performance evaluations, the form classification achieved a mean F1-score of 98%, while the repetition-counting algorithm performed marginally better than existing commercial apps. The estimated average repetition time and range of motion had errors on the order of 0.05 s and 2 degrees, respectively. During a user survey, it was confirmed that the exercise features are both highly accurate and useful. One potential extension could be the integration of automatically generated workouts with repetition and weight targets based on individual workout history and performance, allowing users to select pre-designed workout plans based on their fitness goals and preferences. Additionally, the app could be extended to include social features, such as the ability to connect with friends and share workout data, compete in challenges, or receive motivational messages from trainers or peers. We would also look to train the algorithm on a larger dataset with a diverse set of body types and exercise movements to improve its generalizability. Finally, an interesting direction for future work could be to investigate the use of the app in a clinical or rehabilitation setting, where the accurate measurement of resistance training performance is crucial for recovery and injury prevention. The app could be tailored to specific rehabilitation protocols and used in conjunction with physical therapy to provide patients with objective feedback on their progress.

## Figures and Tables

**Table 1:** Exercise features.

Exercise	Feature	Axis	Used to Identify
Bicep curl	Maximum acceleration	X	The speed of the repetition.
	Gravity height	X	The range of the repetition.
	Rotation symmetry	Y, Z	If a bicep curl is occurring.
Lateral raise	Maximum rotation	Y	The speed of the repetition.
	Gravity height	X	The range of the repetition.
	Minimum roll	N/A	Whether the wrist had been by the user’s side.
	Roll height	N/A	The range of the repetition.
	Gravity/Roll turning points	X, Z	A smooth and repetitive rotation pattern.

**Table 2:** Performance of form classification.

Exercise	Form	Precision	Recall	F1-Score
Bicep curl	Bad range	1.00	1.00	1.00
	Good	1.00	1.00	1.00
	Other	1.00	1.00	1.00
	Too fast	1.00	1.00	1.00
	Avg/Total	1.00	1.00	1.00
Lateral raise	Bad range	0.99	0.99	0.99
	Good	1.00	0.99	1.00
	Other	0.99	1.00	1.00
	Too fast	1.00	1.00	1.00
	Avg/Total	1.00	1.00	1.00
Shoulder press	Bad range	0.89	0.92	0.90
	Good	0.91	0.88	0.90
	Other	1.00	1.00	1.00
	Leg momentum	1.00	1.00	1.00
	Avg/Total	0.95	0.95	0.95

**Table 3:** Performance of repetition counting. Deviations from the ground are shown in bold.

Exercise	Reps	Reps (LEAN)	Reps (GWT)
Bicep curl	8	8	8
	10	10	**11**
	12	12	12
Lateral raise	8	8	**9**
	10	10	10
	12	12	12
Shoulder press	8	**7**	8
	10	10	**11**
	12	**11**	12

**Table 4:** Performance of exercise metrics.

Exercise	Values Estimated Using Video Footage		Values Recorded by LEAN	
	Average Rep Time (s)	Range of Motion (deg)	Average Rep Time (s)	Range of Motion (deg)
Bicep curl	3.06	155	3.02	158.18
Lateral raise	4.12	107	4.18	108.58
Shoulder press	4.20	N/A	4.27	N/A

**Table 5:** Performance of form classification and repetition counting with volunteers. Deviations from the ground are shown in bold.

Exercise	Volunteer 1				Volunteer 2				Volunteer 3			
	Exercise Performance		App Feedback		Exercise Performance		App Feedback		Exercise Performance		App Feedback	
	Form	Reps	Form	Reps	Form	Reps	Form	Reps	Form	Reps	Form	Reps
	Workout 1											
Bicep curl	Good	10	Good	10	Good	10	Good	10	Good	10	Good	10
Lateral raise	Good	10	Good	10	Good	10	Good	10	Good	10	**Bad range**	10
Shoulder press	Good	10	Good	**9**	Good	10	Good	10	Good	10	Good	10
	Workout 2											
Bicep curl	Good	10	Good	10	Good	10	Good	10	Good	10	Good	10
Lateral raise	Good	10	Good	10	Good	10	**Bad range**	**9**	Good	10	**Bad range**	10
Shoulder press	Good	10	Good	**9**	Good	10	Good	10	Good	10	Good	**9**
	Workout 3											
Bicep curl	Bad range	10	Bad range	10	Too fast	10	Too fast	10	Too fast	10	Too fast	10
Lateral raise	Bad range	10	Bad range	10	Bad range	10	Bad range	**9**	Too fast	10	Too fast	10
Shoulder press	Bad range	10	Bad range	**9**	Bad range	10	**Good**	10	Bad range	10	Bad range	10

**Table 6:** Results from user survey during acceptance testing.

	Strongly Agree	Agree	Neither Agree Nor Disagree	Disagree	Strongly Disagree
The iPhone app was easy to understand and navigate.	2	4	0	0	0
The Apple Watch app was easy to understand and navigate.	3	1	0	0	0
The form analysis feature was useful in identifying poor form and how to correct it.	1	2	1	0	0
The form analysis feature was accurate.	3	1	0	0	0
The repetition counting feature was useful.	1	2	0	1	0
The repetition counting feature was accurate.	0	2	2	0	0
The exercise metrics were useful and/or interesting.	2	2	0	0	0

## Data Availability

The code and the data for building the app and for recreating the performance tests are available at https://github.com/Resistance-Training-App (accessed on 1 March 2023).
